# Comparison of two common aEEG classifications for the prediction of neurodevelopmental outcome in preterm infants

**DOI:** 10.1007/s00431-016-2816-5

**Published:** 2016-12-06

**Authors:** Nora Bruns, Frauke Dransfeld, Britta Hüning, Julia Hobrecht, Tobias Storbeck, Christel Weiss, Ursula Felderhoff-Müser, Hanna Müller

**Affiliations:** 1 Department of Pediatrics I, Neonatology, Pediatric Intensive Care, Pediatric Neurology, University Hospital Essen, University Duisburg-Essen, Hufelandstr. 55, 45147 Essen, Germany; 2 Institute of Medical Statistics and Biomathematics, University Hospital Mannheim,, University of Heidelberg, Ludolf-Krehl-Straße 13-17D, 68167 Mannheim, Germany; 3 Division of Neonatology, Department of Pediatrics, University of Heidelberg, Im Neuenheimer Feld 430, 69120 Heidelberg, Germany

**Keywords:** Preterm infant, aEEG, Amplitude-integrated EEG, Neurodevelopmental outcome, Mortality

## Abstract

Neurodevelopmental outcome after prematurity is crucial. The aim was to compare two amplitude-integrated EEG (aEEG) classifications (Hellström-Westas (HW), Burdjalov) for outcome prediction. We recruited 65 infants ≤32 weeks gestational age with aEEG recordings within the first 72 h of life and Bayley testing at 24 months corrected age or death. Statistical analyses were performed for each 24 h section to determine whether very immature/depressed or mature/developed patterns predict survival/neurological outcome and to find predictors for mental development index (MDI) and psychomotor development index (PDI) at 24 months corrected age. On day 2, deceased infants showed no cycling in 80% (HW, *p* = 0.0140) and 100% (Burdjalov, *p* = 0.0041). The Burdjalov total score significantly differed between groups on day 2 (*p* = 0.0284) and the adapted Burdjalov total score on day 2 (*p* = 0.0183) and day 3 (*p* = 0.0472). Cycling on day 3 (HW; *p* = 0.0059) and background on day 3 (HW; *p* = 0.0212) are independent predictors for MDI (*p* = 0.0016) whereas no independent predictor for PDI was found (multiple regression analyses).

*Conclusion*: Cycling in both classifications is a valuable tool to assess chance of survival. The classification by HW is also associated with long-term mental outcome.

**Table:** 

**What is Known:** •*Neurodevelopmental outcome after preterm birth remains one of the major concerns in neonatology.* *•aEEG is used to measure brain activity and brain maturation in preterm infants.*
**What is New:** •*The two common aEEG classifications and scoring systems described by Hellström-Westas and Burdjalov are valuable tools to predict neurodevelopmental outcome when performed within the first 72 h of life.* *•Both aEEG classifications are useful to predict chance of survival. The classification by Hellström-Westas can also predict long-term outcome at corrected age of 2 years.*

## Introduction

Preterm birth accounts for 11% of all live-births worldwide and is increasing in most countries [4]. In recent years, survival rates of even the most immature preterm infants have markedly improved. Nonetheless, these infants are still at risk for neurologic sequelae that extend beyond the neonatal period [21]. About one fourth of survivors of extremely preterm birth suffer from relevant neurological impairment [9]. Adverse functional consequences persist into adolescence and early adulthood and pose a growing social and economic burden on families and society [1, 13, 16, 20]. Strong efforts are being made to identify markers for early prediction of motor and cognitive outcome in order to aid parental counseling, initiate early support, and thereby improve long-term morbidity.

Originally being developed as a bedside monitor in adult intensive care [15], the use of amplitude-integrated EEG (aEEG) has become more and more widespread in neonatal intensive care units (NICU) for continuous monitoring of cerebral function in neonates in recent years [19]. The main field of clinical use of aEEG is the prediction of cerebral outcome after birth asphyxia in term infants [2, 12, 22], the detection of cerebral seizure activity and the surveillance of antiepileptic drug treatment [11].

Also in the population of preterm infants, aEEG is increasingly being used. It is generally well accepted by the NICU staff and has been proven to be a safe method for cerebral function monitoring even in extremely preterm infants [7]. Recent studies have shown a correlation between aEEG recordings obtained during the first weeks of life and short- and long-term neurodevelopmental outcome of preterm infants [3, 14, 18, 25, 27, 29]. In order to become a practical tool for clinical routine use, it is necessary to find easy-to-use schemes for assessment of aEEG tracings in preterm infants.

Electrocortical activity, and thus the aEEG background pattern, depends on the infant’s gestational age [11]. Several authors have made suggestions for classification and scoring of aEEGs. One of the first publications for the classification of background patterns, sleep-wake cycling and seizure activity in preterm and newborn infants was published by Hellström-Westas et al. [11]. Burdjalov et al. made an attempt to quantify brain maturation by aEEG between 24 and 39 weeks with a scoring system [6]. This score has been cited and modified by several authors describing maturational changes of electrocortical function in premature infants [6, 11, 17, 23, 24]. Another system using reference values for the corresponding gestational age was developed by Olischar et al. [17]. Later, these reference values were used to build a score based on the combination of background activity, sleep-wake cycling, and seizure activity [14]. In recent years, several studies correlated aEEG patterns with clinical conditions (e.g., patent ductus arteriosus, small for gestational age) [5, 10]. Electronical assessment of aEEG is possible but less common, even though a few publications used this approach [5, 26, 28].

The aim of our study was to compare two common classifications and scoring systems (Hellström-Westas and Burdjalov), regarding their value for the prediction of survival and of mental and psychomotor outcome. The hypothesis of our study is that survival and long-term outcome following preterm birth may be predicted by early aEEG recordings. Therefore, in our single center cohort of non-sedated infants, we investigated the correlation between aEEG parameters as defined by two common scores obtained within the first 72 h of life and outcome as quantified by survival and by the Bayley II testing at 24 months corrected age. Survival and long-term outcome following preterm birth can be predicted by early aEEG recordings. Both methods proved to be valuable for the assessment of aEEG tracings.

## Methods

### Patient recruitment

**Fig. 1:**
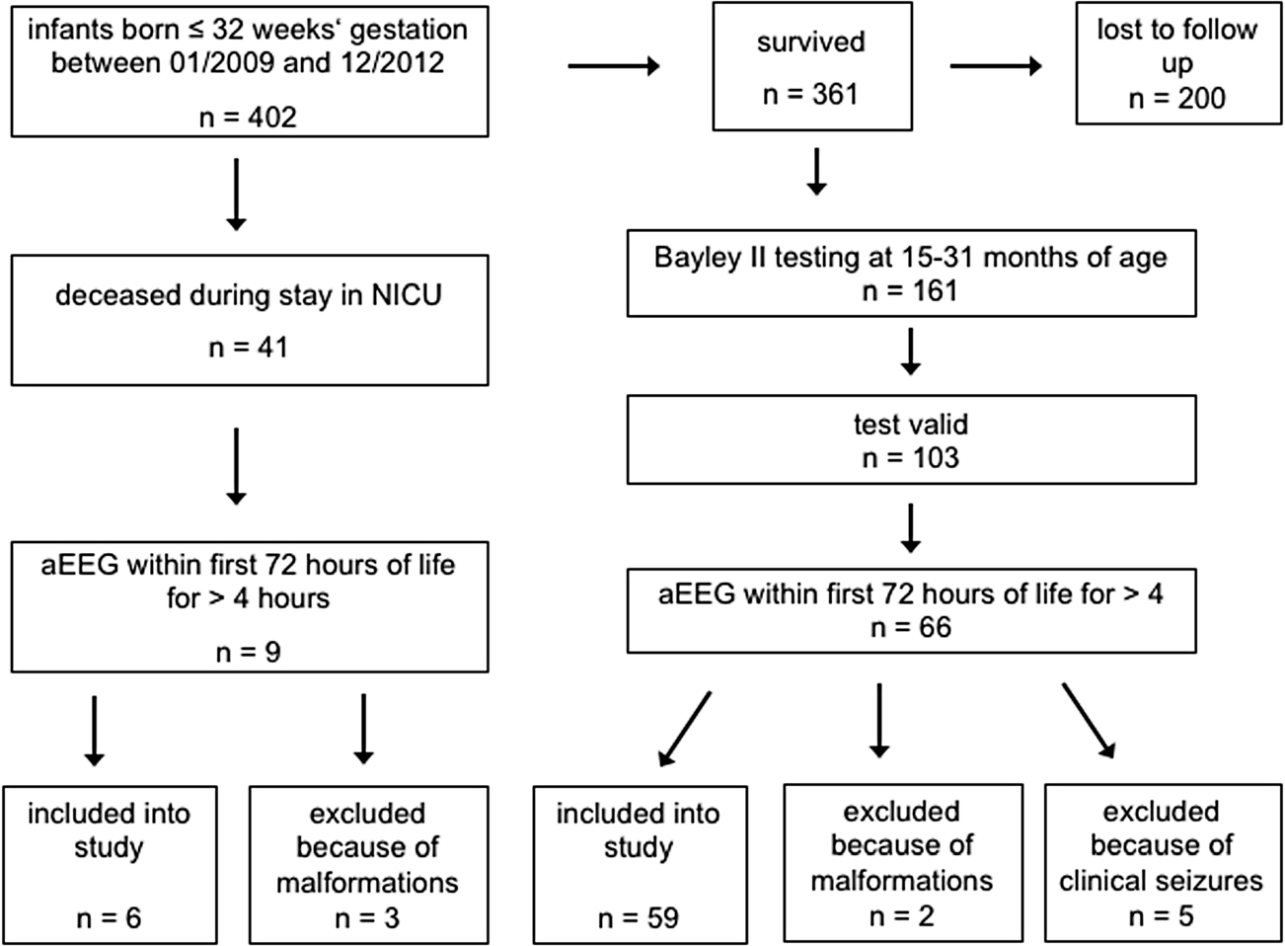
Patient recruitment

All preterm infants (gestational age ≤ 32 weeks) who were treated in our unit between 01/2009 and 12/2012 and received both aEEG recordings of at least 4 h duration within 72 h of life and the Bayley II at 24 months corrected age or died during their stay in the NICU were eligible for our retrospective study. From a total of 402 patients born ≤32 weeks gestational age, 65 infants were included into the study (Fig. 1). Gestational age was assessed based on the best obstetric estimate (the first day of the last menstruation and ultrasound). Sedation or opiate medication within 12 h before or during the recording was an exclusion criterion. Between 06/2009 and 10/2010, infants were recruited for aEEG recordings as part of the NEOBRAIN study, which was a European multicenter prospective trial. The study included infants <28 weeks gestational age, but in our center, we sought informed consent for all infants ≤32 weeks gestational age in order to conduct aEEG tracings. After the end of recruiting for NEOBRAIN, aEEGs became part of the clinical routine in our NICU. For infants born after 10/2010, parents signed a written consent that clinical data may be used for scientific retrospective analysis upon admission of each infant. The study was approved by the local ethics committee and in accordance with the 1964 Helsinki declaration and its later amendments or comparable ethical standards.

### aEEG recording

All aEEGs were recorded by the nurse or the doctor in charge of the infant within the first 72 h of life. The aEEG was recorded as a two-channel EEG using needle electrodes or gold caps with BRM2 and BRM3 monitors (BrainZ Instruments, New Zealand). Electrodes were placed on the scalp corresponding to the positions C3, P3, C4, and P4 of the international 10–20 system. A reference electrode was placed on the back of the infant.

### aEEG interpretation

All aEEGs were analyzed by two independent investigators (N.B. and H.M.) blinded for the patients’ outcome and using predefined criteria [6, 11]. The first section with continuously good quality of tracing (no artifacts, impedance <15 kΩ) for 4 h within each day of life was selected for assessment. The aEEG recordings were independently scored by each person. In case of disagreeing scores, the tracing was reassessed by both investigators together and consent was sought. The strength of interrater agreement was good (Hellström-Westas: weighted *κ* = 0.7589 (CI 0.7177, 0.8001); Burdjalov: weighted *κ* = 0.6265 (CI 0.5626, 0.6904)).

### aEEG classifications

For the assessment of the Burdjalov score, we evaluated the tracings following the criteria from the original publication [6]. The total score adds up from visual classification of four criteria: continuity, the expression of sleep-wake cycling (SWC), the amplitude of the lower border (LBA), and the bandwidth (BW). The maximum score is 13 and should be reached at a corrected gestational age of 36/37 weeks. Additionally, we adapted the Burdjalov total score (see Table 1) and calculated an “adapted Burdjalov total score”. Furthermore, we assessed the recordings following the classification by Hellström-Westas which additionally includes criteria for pathologic findings [11].

**Table 1:** Overview over parameters for interpretation of aEEG

	Very immature/depressed pattern	More mature/developed pattern
Hellström-Westas background pattern	Burst suppression pattern/flat trace	Discontinuous/continuous pattern
Hellström-Westas sleep-wake cycling	No cycling	Imminent/immature cycling
Burdjalov continuity	Discontinuous (^a^0 point; ^b^0 point)	Somewhat continuous or better (^a^1 to 2 points; ^b^1 point)
Burdjalov sleep-wake cycling	No cycling (^a^0 point; ^b^0 point)	Waves first appear or better (^a^1 to 5 points; ^b^1 point)
Burdjalov lower border	< 5 μV (^a^0 to 1 point; ^b^0 point)	>5 μV (^a^2 to 4 points; ^b^1 point)
Burdjalov bandwidth	Very depressed/very immature (^a^0 to 1 point; ^b^0 point)	Immature or better (^a^2 to 4 points; ^b^1 point)

^ab^Burdjalov total score (according to [6]), adapted Burdjalov total score

### Outcome and Bayley II testing

Testing was performed by trained staff as part of the national routine follow-up program for preterm infants. MDI scores were determined in 59 infants and PDI scores in 53 infants. Six of the 65 included infants died during their stay in the NICU, thus no MDI and PDI scores were available. Favorable outcome was defined as both MDI and PDI ≥ 70, poor outcome was defined either one or both MDI and PDI < 70. Infants who died during the neonatal period were summed up in a third group. MDI or PDI scores < 50 were considered as 45 to enable statistical analysis.

### Statistical analysis

For quantitative variables, mean and standard error of the mean (SEM) have been calculated; ordinal scaled parameters are presented by median and range; for qualitative factors, absolute and relative frequencies are given. To correlate the outcome (good–poor–dead) with aEEG parameters, findings of each parameter were divided into two categories: very immature/depressed versus more mature/developed (Table 1).

For each day, we evaluated whether aEEG parameters showed a significant difference between the groups using the chi-square-test, Fisher’s exact test, Kruskal-Wallis, or Mann Whitney *U* test, as appropriate. In order to compare two mean values resulting from data which are approximately normally distributed, two sample *t* tests has been performed. In order to compare three outcome groups regarding gestational age and birth weight, a one-way ANOVA has been performed followed by the Tukey-Kramer post hoc tests in the case of a significant test result. To evaluate the association between aEEG parameters and MDI/PDI, we performed multiple regression analyses adjusting for birth weight and gestational age.

The strength of interrater agreement concerning aEEG recordings has been assessed using weighted Kappa coefficients using weight factors according to Cicchetti Allison [8].

All statistical calculations have been done using SAS software, release 9.3 (SAS Institute Inc., Cary, NC, USA). The result of a statistical test has been considered as significant for *p* < 0.05.

## Results

Sixty-five infants met the inclusion criteria. Mean birth weight was 1022 ± 47 g (mean ± SEM; range 440–1880 g; median 985 g) with a mean gestational age of 27.3 ± 0.3 weeks (mean ± SEM; range 23–32 weeks; median 27 weeks). Six infants died during treatment in our NICU. For the clinical details of the cohort, we refer to Tables 2 and 3. The median aEEG recording time was 63 h and 41 min (range 4:55–91:16) with 50 utilizable aEEGs on day 1, 61 on day 2, and 52 on day 3. Bayley testing was performed in the surviving infants at a mean age of 24.0 ± 0.5 months (mean ± SEM), (range 15–31 months, quartiles 23 and 27 months).

**Table 2:** Clinical data of cohort

Outcome	GA: mean ± SEM (range; median) [weeks]	BW: mean ± SEM (range; median) [g]	Gender [male/female]	CA [n]	SGA <10. P. [n]	Caffeine therapy [n]	Indo-methacin therapy [n]	IVH III/IV [n]	PVL [n]	Severe BPD^a^ [n]	Mechanical ventilation within first 72 h of life [n]	Catech within first 72 h of life [n]
Good (*n* = 40)	28.1 ± 0.3 (24–32; 28.0)	1125 ± 52 (610–1880; 1060)	19/21	9	4	39	17	1	1	2	9	3
Poor (*n* = 19)	26.7 ± 0.6 (23–31; 27.0)	959 ± 95 (450–1810; 985)	5/14	5	3	18	13	0	2	6	11	0
Dead (*n* = 6)	23.7 ± 0.3 (23–25; 24.0)	534 ± 33 (440–649; 523)	0/6	1	1	6	1	4	0	–	6	0
Total (*n* = 65)	27.3 ± 0.3 (23–32; 27.0)	1022 ± 47 (440–1880; 985)	24/41	15	8	63	32	5	3	8	26	3

*GA* gestational age, *BW* birth weight, *CA* chorioamnionitis, *SGA* small for gestational age, *BPD* bronchopulmonary dysplasia, *catech* catecholamines

^a^O2 > 36 weeks postmenstrual age; mechanical ventilation within first 72 h of life inludes only ventilation longer than 1 h

**Table 3:** Clinical data of deceased infants

No.	GA [weeks]	BW [g]	Umbilical arterial blood pH	APGAR ’10	Day of life when died	Cause of death
1	23	470	Not determinable	7	3	Severe IVH, respiratory failure
2	23	495	7.41	7	7	Septic shock
3	24	610	7.29	8	4	Cardiorespiratory failure
4	24	440	7.29	8	9	Multi organ failure
5	23	550	6.60	3	2	Cardiorespiratory failure, asphyxia after placentar disruption
6	25	640	7.21	7	9	Severe IVH, intestinal perforation, cardiorespiratory failure

*GA* gestational age, *BW* birth weight, *IVH* intraventricular hemorrhage

**Fig. 2:**
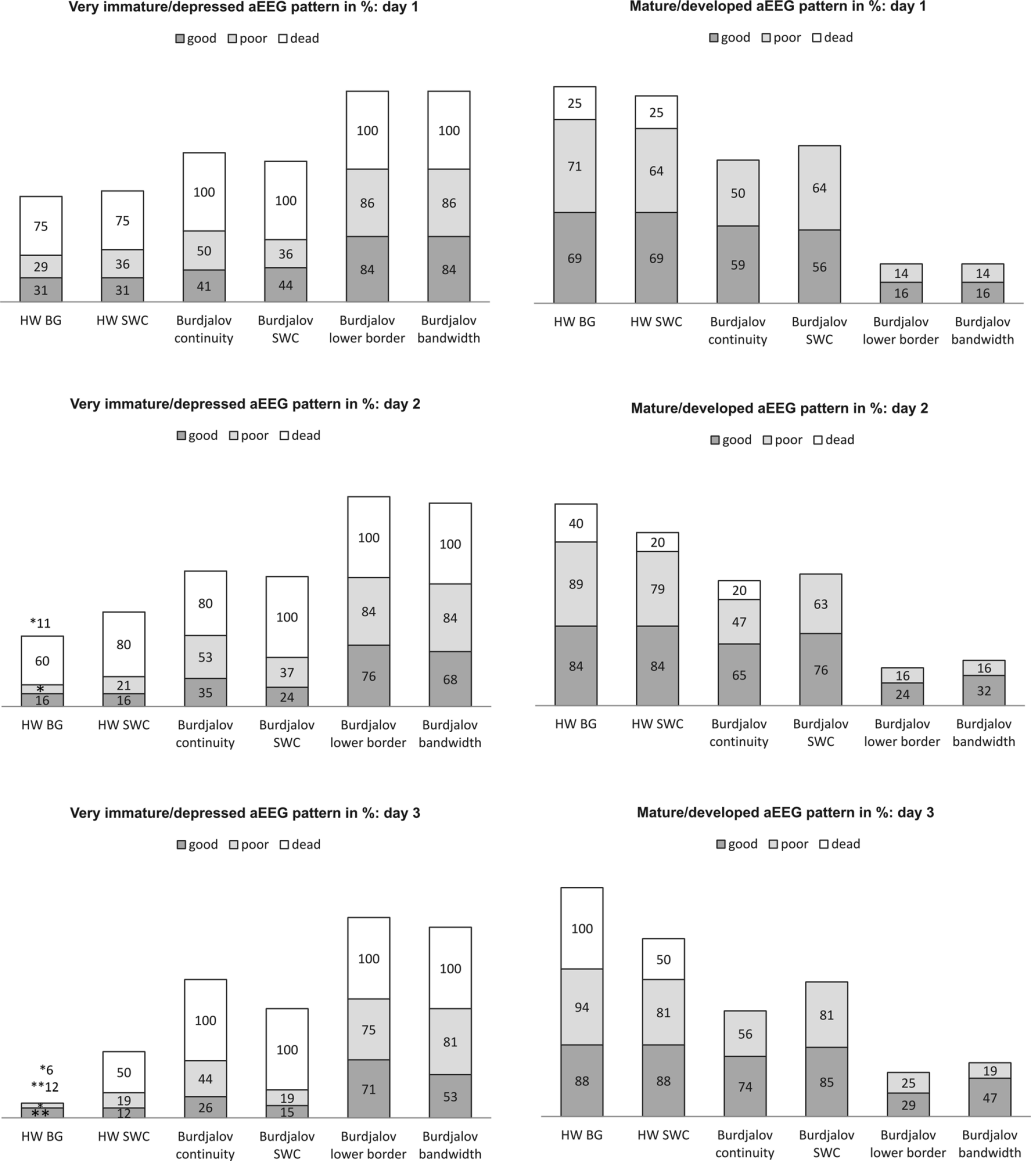
Distribution of very immature/depressed (*left side*) and of mature/developed (*right side*) aEEG patterns on each day in all three outcome groups (good outcome–poor outcome–dead) is demonstrated in percent of each outcome group. Adverse short-term outcome (death) is reflected by the absence of mature/developed patterns during the first 72 h of life. For each day, we evaluated whether aEEG parameters showed a significant difference between groups (good–poor–death) using Fisher’s exact test: Cycling on day 2 (Hellström-Westas, *p* = 0.0140; Burdjalov, *p* = 0.0041) was significant; for details see Table 4

The first day of life: The distribution of background patterns and sleep-wake cycling was very similar between the groups with good and poor outcome following both the definitions of Hellström-Westas and Burdjalov. However, in the group of deceased infants very immature/depressed findings for both background pattern and cycling were more prevalent, but reached no statistical significance (Fig. 2, see Table 4 for *p* values).

**Table 4:** aEEG patterns in the different outcome groups on day 1–3

		Day 1 in all outcome groups				Day 2 in all outcome groups				ay 3 in all outcome groups			
		Good (*n* = 32)	Poor (*n* = 14)	Dead (*n* = 4)	*p* value	Good (*n* = 37)	Poor (*n* = 19)	Dead (*n* = 5)	*p* value	Good (*n* = 34)	Poor (*n* = 16)	Dead (*n* = 2)	*p* value
HW background	In % (n): pathological (burst suppression/flat trace) vs. discontinuous/continuous	31 (10) vs 69 (22)	29 (4) vs 71 (10)	75 (3) vs 25 (1)	0.2548 ^a^	16 (6) vs 84 (31)	11 (2) vs 89 (17)	60 (3) vs 40 (2)	0.0567 ^a^	12 (4) vs 88 (30)	6 (1) vs 94 (15)	0 (0) vs 100 (2)	1.000 ^a^
HW cycling	In % (n): no cycling vs. immature/mature	31 (10) vs 69 (22)	36 (5) vs 64 (9)	75 (3) vs 25 (1)	0.2744 ^a^	16 (6) vs 84 (31)	21 (4) vs 79 (15)	80 (4) vs 20 (1)	0.0140 ^a^	12 (4) vs 88 (30)	19 (3) vs 81 (13)	50 (1) vs 50 (1)	0.2632 ^a^
Burdjalov continuity	In % (n): discontinuous vs. somewhat continuous/continuous	41 (13) vs 59 (19)	50 (7) vs 50 (7)	100 (4) vs 0 (0)	0.0851 ^a^	35 (13) vs 65 (24)	53 (10) vs 47 (9)	80 (4) vs 20 (1)	0.1260 ^a^	26 (9) vs 74 (25)	44 (7) vs 56 (9)	100 (2) vs 0 (0)	0.0876 ^a^
Burdjalov cycling	In % (n): no cycling vs. waves first appear or better	44 (14) vs 56 (18)	36 (5) vs 64 (9)	100 (4) vs 0 (0)	0.0931 ^a^	24 (9) vs 76 (28)	37 (7) vs 63 (12)	100 (5) vs 0 (0)	0.0041 ^a^	15 (5) vs 85 (29)	19 (3) vs 81 (13)	100 (2) vs 0 (0)	0.0504 ^a^
Burdjalov lower border	In % (n): <5 μV vs. >5 μV	84 (27) vs 16 (5)	86 (12) vs 14 (2)	100 (4) vs 0 (0)	1.000 ^a^	76 (28) vs 24 (9)	84 (16) vs 16 (3)	100 (5) vs 0 (0)	0.6146 ^a^	71 (24) vs 29 (10)	75 (12) vs 25 (4)	100 (2) vs 0 (0)	1.000 ^a^
Burdjalov bandwidth	In % (n): very depressed/very immature vs. immature or better	84 (27) vs 16 (5)	86 (12) vs 14 (2)	100 (4) vs 0 (0)	1.000 ^a^	68 (25) vs 32 (12)	84 (16) vs 16 (3)	100 (5) vs 0 (0)	0.2327 ^a^	53 (18) vs 47 (16)	81 (13) vs 19 (3)	100 (2) vs 0 (0)	0.0810 ^a^
Burdjalov total score ^d^	Score: range; median	1–8; 3.5	1–9; 3.5	0–2; 2	0.0871 ^b^	1–10; 4.0	2–10; 3.0	2–3; 3.0	0.0284 ^b^	1–9; 5.0	2–9; 4.0	2.0; 2.0	0.0526 ^b^
Burdjalov total score (adapted ^e^)	Score: range; median	0–4; 1.5	0–4; 1.5	0–0; 0	0.0769 ^b^	0–4: 2.0	0–4; 1.0	0–1; 0	0.0183 ^b^	0–4; 2.0	0–4; 2.0	0–0; 0	0.0472 b
Birth weight	[g] mean ± SEM (range)	1112 ± 56 (610–1730)	976 ± 121 (450–1810)	524 ± 37 (440–610)	0.0007 ^c^	1134 ± 54 (610–1880)	959 ± 95 (450–1810)	531 ± 40 (440–640)	0.0018 ^c^	1102 ± 59 (610–1880)	889 ± 93 (450–1490)	625 ± 15 (610–640)	0.0447 ^c^
Gestational age	[weeks] mean ± SEM (range)	27.9 ± 0.4 (24.0–32.0)	26.9 ± 0.7 (23.0–31.0)	23.5 ± 0.3 (23.0–24.0)	<0.0001 ^c^	28.1 ± 0.4 (24.0–32.0)	26.7 ± 0.6 (23.0–31.0)	23.8 ± 0.4 (23.0–25.0)	0.0004 ^c^	27.8 ± 0.4 (24.0–32.0)	26.3 ± 0.6 (23.0–30.0)	24.5 ± 0.5 (24.0–25.0)	0.0238 ^c^

*HW* Hellström-Westas, *vs* versus, *SEM* standard error of the mean

^a^Fisher’s exact test, ^b^ Kruskal-Wallis test, ^c^ one-way ANOVA, ^d^ Burdjalov total score according to [6], ^e^ adapted Burdjalov total score (see Table 1)

There was a statistical difference when comparing the birth weights or gestational age between the three outcome groups (gestational age *p* < 0.0001; birth weight *p* = 0.0007 using one-way ANOVAs). Post hoc tests according to Tukey-Kramer revealed significant differences between good and bad outcome (*p* < 0.0001 and *p* = 0.0006, accordingly) as well as between poor and bad outcome (*p* = 0.0140 and *p* = 0.0278, accordingly). The differences between good and poor outcome failed to be significant (*p* = 0.0597 and 0.2028, accordingly)

Second day of life: 16–21% of infants with good and poor outcome each presented very immature/depressed findings in cycling (Hellström-Westas), whereas 24% of the infants with good and 37% of infants with poor outcome showed very immature/depressed characteristics in cycling by Burdjalov. Again, the group of deceased infants showed very immature/depressed patterns in a higher percentage of cases: no cycling could be found in 80% (Hellström-Westas) and 100% (Burdjalov). The difference for both parameters was significant (*p* = 0.0140 and *p* = 0.0041; Fig. 2, Table 4). There was no significance for background pattern (Hellström-Westas), continuity, lower border, and bandwidth (all Burdjalov).

Third day of life: On day 3, we found no difference between aEEG parameters, but the absolute number in the group of deceased infants was small (*n* = 2). For the details for all 3 days of life refer to Table 4.

**Fig. 3:**
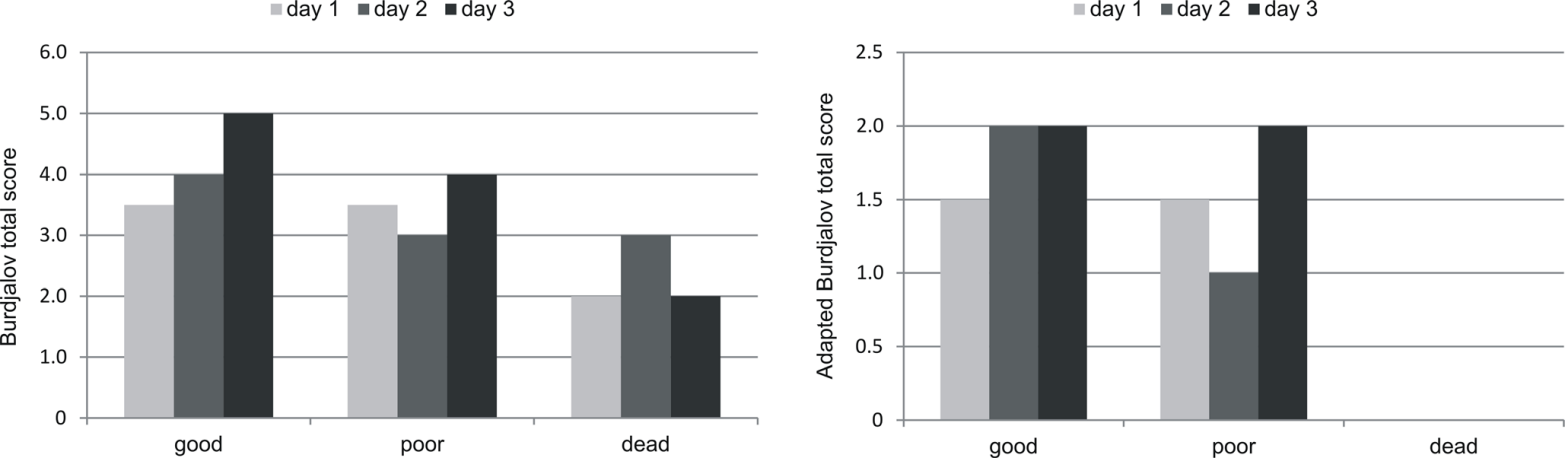
The Burdjalov total score and the adapted Burdjalov total score (median) in the three different outcome groups during the first 3 days of life the Burdjalov total score significantly differed between outcome groups on day 2 (*p* = 0.0284, Kruskal-Wallis test) and the adapted Burdjalov total score on day 2 (*p* = 0.0183, Kruskal-Wallis test) and day 3 (*p* = 0.0472, Kruskal-Wallis test), see also Table 4

The Burdjalov total score significantly differed between groups on day 2 (*p* = 0.0284, day 1 and 3 not significant) and the adapted Burdjalov total score on day 2 (*p* = 0.0183) and day 3 (*p* = 0.0472). However, the range is large and overlaps between groups, but the maximum total score is lower in the group of deceased infants (Table 4, Fig. 3).

Multiple regression analyses revealed sleep-wake cycling (Hellström-Westas) on day 3 of life (*p* = 0.0059) and background on day 3 (Hellström-Westas; *p* = 0.0212) as independent predictors for MDI whereas no independent predictor for PDI was found (multiple regression analyses).

## Discussion

Continuous monitoring of brain function in very immature infants remains exceptional in clinical routine, as recording, assessment and interpretation of preterm EEG and aEEG are time consuming and appear sometimes conflicting with principles of minimal handling. To establish easy algorithms for aEEG interpretation, several authors have reported maturational changes of electrocortical activity according to both postnatal and gestational age. Recent studies have pointed out a correlation of early aEEG parameters with short- and long-term neurological outcome [3, 14, 18, 25, 27, 29]. In this study, we refer to and compare the classifications by Hellström-Westas et al. [11] and the aEEG maturation score introduced by Burdjalov et al. in a single center cohort of prematurely born infants ≤32 weeks gestation [6]. We showed that there is a correlation in both classifications between the absence of cycling on the second day of life and the risk of death. Also, we demonstrated a correlation of background pattern and cyclicity (both HW) with long-term mental outcome.

It has previously been shown, that continuity within the first 72 h of life, especially the presence of pathological background pattern correlates with adverse short-term outcome (IVH °III/IV) in infants between 25 and 32 weeks gestational age [25]. The presence of low voltage pattern and the lack of sleep-wake cycling during the first 72 h of life were associated with death in preterm infants ≤1500 g or ≤32 weeks [3]. The present results from our cohort are in wide agreement with these findings. Infants who died during treatment on the NICU rarely showed physiological patterns in aEEG recordings within the first 72 h of life. The Burdjalov total score and the adapted Burdjalov total score did not add additional information despite the fact that significant differences between scores were found on day 2 (usual Burdjalov total score) or on days 2 and 3 (adapted Burdjalov total score), as the ranges between the different outcome groups were strongly overlapping.

There is strong evidence that neuropsychological deficits can be predicted within the first 72 h of life. Sleep-wake cycling and a combined score of background activity, cycling and seizure activity during the first 2 weeks of life correlate with Bayley results at 3 years of age, cerebral palsy and death in preterm infants <30 weeks gestational age [14]. Prolonged interburst intervals and burst suppression pattern are strong predictors of poor neurodevelopmental outcome at 2 years corrected age in preterm infants between 22 and 30 gestational weeks [29]. Burdjalov total scores from aEEGs obtained within the first 6 weeks of life in infants ≤30 gestational weeks were associated with perinatal factors that are known to predict adverse neurodevelopmental outcome [18]. On the other hand, the rate of brain wave maturation between 28 and 36 weeks gestational age in preterm infants <28 weeks does not predict neurological development at 18 to 22 months corrected age [27].

The majority of studies on prediction of outcome by aEEG revealed, that the presence of pathological patterns predicts poor outcome (short- and long-term). Our findings are in agreement with the fact that prediction of favorable outcome seems to be more complex. Of those infants who died during their stay in the NICU, only very few showed physiological aEEG patterns. Thus, the presence of physiological patterns seems to be a predictor of survival. Only the classification by Hellström-Westas included independent predictors for long-term outcome: Cycling and background on day 3 were associated with MDI at 24 months corrected age.

We conclude that both systems are valuable tools for the assessment of aEEG tracings in preterm infants. They are easy to apply and make bedside evaluation possible, as they can be interpreted “at a glance”. One main difference between the two classifications is that the Burdjalov score is primarily designed to describe the physiological maturation of electrocortical activity and only indirectly provides measures for pathological patterns. The classification by Hellström-Westas on the other hand, is designed to distinguish pathological and physiological patterns rather than to describe maturational changes over time. In our study, the presence of cycling was helpful to predict survival. Background pattern and cycling (both HW) were helpful to predict long-term mental outcome. As the results of this study are preliminary data, prospective studies are needed to further evaluate the potential contribution of early aEEG recordings to decision-making in very sick preterm infants.

## Abbreviations

aEEG: Amplitude-integrated EEG
BG: Background pattern
BW: Bandwidth
EEG: Electroencephalography
HW: Hellström-Westas
IVH: Intraventricular hemorrhage
LBA: Amplitude of lower border
MDI: Mental development index
PDI: Psychomotor development index
SEM: Standard error of the mean
SWC: Sleep-wake cycling
